# User training for machine learning controlled upper limb prostheses: a serious game approach

**DOI:** 10.1186/s12984-021-00831-5

**Published:** 2021-02-12

**Authors:** Morten B. Kristoffersen, Andreas W. Franzke, Raoul M. Bongers, Michael Wand, Alessio Murgia, Corry K. van der Sluis

**Affiliations:** 1 Department of Rehabilitation Medicine, University of Groningen, University Medical Center Groningen, Groningen, Netherlands; 2 Department of Human Movement Sciences, University of Groningen, University Medical Center Groningen, Groningen, Netherlands; 3 IDSIA, USI & SUPSI, Manno-Lugano, Switzerland

**Keywords:** Serious games, Machine learning, Prosthesis, Motor learning, EMG, Structured training

## Abstract

**Background:**

Upper limb prosthetics with multiple degrees of freedom (DoFs) are still mostly operated through the clinical standard Direct Control scheme. Machine learning control, on the other hand, allows controlling multiple DoFs although it requires separable and consistent electromyogram (EMG) patterns. Whereas user training can improve EMG pattern quality, conventional training methods might limit user potential. Training with serious games might lead to higher quality EMG patterns and better functional outcomes. In this explorative study we compare outcomes of serious game training with conventional training, and machine learning control with the users’ own one DoF prosthesis.

**Methods:**

Participants with upper limb absence participated in 7 training sessions where they learned to control a 3 DoF prosthesis with two grips which was fitted. Participants received either game training or conventional training. Conventional training was based on coaching, as described in the literature. Game-based training was conducted using two games that trained EMG pattern separability and functional use. Both groups also trained functional use with the prosthesis donned. The prosthesis system was controlled using a neural network regressor. Outcome measures were EMG metrics, number of DoFs used, the spherical subset of the Southampton Hand Assessment Procedure and the Clothespin Relocation Test.

**Results:**

Eight participants were recruited and four completed the study. Training did not lead to consistent improvements in EMG pattern quality or functional use, but some participants improved in some metrics. No differences were observed between the groups. Participants achieved consistently better results using their own prosthesis than the machine-learning controlled prosthesis used in this study.

**Conclusion:**

Our explorative study showed in a small group of participants that serious game training seems to achieve similar results as conventional training. No consistent improvements were found in either group in terms of EMG metrics or functional use, which might be due to insufficient training. This study highlights the need for more research in user training for machine learning controlled prosthetics. In addition, this study contributes with more data comparing machine learning controlled prosthetics with Direct Controlled prosthetics.

## Background

In recent years, multiarticulate myoelectric prosthetic hands controlled using machine learning (ML) algorithms have been brought from the labs to the clinics by both commercial enterprises [1–3] and research groups [4–8]. ML control can reveal the full potential of multiarticulate hands, as the clinical standard two-site “Direct Control” (DC) scheme is currently one of the main limiting factors in prosthetic control. In contrast to DC, ML control does not require isolated subsequent electromyographic (EMG) signals derived from two electrodes to control all available grip modes, but utilizes more intuitive control generated by patterns of muscle contractions [9]. However, ML control currently suffers from robustness issues while DC is faster in tasks that only require one degree of freedom (DoF) [4, 6].

The benefit of ML control is that the user does not need to actively switch between DoFs or grips. Unlike DC, ML control is not based on a direct mapping from each muscle to a direction in a DoF: it performs a complex mapping between EMG activity and control commands, thus allowing the user to generate a potentially larger set of control commands. EMG patterns are measured on the residual muscles in the stump using an array of electrodes, commonly between four to eight, then features are calculated from the EMG and used as input for a learning algorithm which maps the features to prosthetic control commands. ML control should lead to the utilisation of at least a few DoFs and grips. Use of more DoFs might reduce compensatory movements of the trunk and shoulders which are areas with many complaints for people with upper limb absence [10]. Therefore, ML control might lead to better function and reduce the risk of pain and further impairment.

Independent of whether DC or ML control is used, user training is required to skilfully control a myoelectric prosthesis. For DC, training focuses on contracting the two muscles used independently from each other and on executing switching commands. For ML control training focuses on adapting the (phantom) movements used for control to achieve the most distinct EMG patterns, and on performing the movements consistently [8, 11, 12]. A coach assists the trainee in selecting how the movement(s) should be adapted by studying the feature space. Such adaptations mostly focus on the non-essential fingers e.g. flexing the little finger while performing a pinch. Adaptations retain the basis of the original movement (e.g. thumb and index finger touching), but by adding the non-essential part (e.g. flexing the little finger) the generated EMG pattern changes and becomes more distinct from other movements. We refer to this training scheme as conventional training. Conventional training has some drawbacks. Firstly, training requires a coach, which restricts training to the clinic leading to increased costs and limited training exposure. Furthermore, coaching relies on declarative statements such as “when doing the pinch, flex the phantom little finger” which induces an internal focus of attention (i.e. focus on body parts) that, compared to an external focus of attention (i.e. focus on the result), may lead to slower and less accurate motor performance as well as increased cognitive effort [13]. Lastly, the possible separation of EMG patterns is limited by using movements that correspond to the actuation of the prosthesis (e.g. phantom fine pinch leads to prosthetic fine pinch). Possible movements which are not prioritised due to limited functional benefits might generate more separable EMG patterns than the movements which resemble the actuation of the prosthesis [14, 15].

As an alternative to conventional training, we suggest the use of serious games. Serious games have as purpose to teach or train a skill while keeping the trainee engaged with entertaining challenges and individualised feedback [16–18]. Other benefits of serious games are that the trainee focuses on the screen which induces an external focus of attention. Furthermore, serious games can facilitate implicit learning as the game gives a safe environment that allows for experimentation so that the trainee can find the best solution to the challenge without being told to explicitly. Serious games have been used to train proportional control and switching grip modes, as commonly used in commercial upper-limb prosthetics [19–21].

We have previously used a serious game to train ML control in able-bodied participants [15]. We found that game training led to more separable EMG patterns than conventional training with coaching, while performance was similar when evaluating real-time classifier performance using a screen-based test. However, since the participants were able-bodied it is unknown if these results are also applicable to individuals with upper limb absence (ULA), since the neuromuscular changes caused by amputation affects the EMG [22, 23]. Furthermore, it is also unknown if and to what extent game performance transfers to functional prosthesis use, as transfer to functional use has not been explicitly tested with serious games and is the most important metric to gauge a new training scheme. The purpose of this exploratory study was to compare game training with conventional training using coaching for individuals with ULA when learning ML based control, using measures of functional prosthesis use. Specifically, we asked if game-based training leads to 1) more separated and consistent EMG patterns, 2) a higher rate of improvement in EMG pattern separability and consistency as result of learning, 3) use of more degrees of freedom and 4) better outcomes in functional prosthesis use, compared to conventional training with coaching. In addition, we performed baseline measurements using the participants’ own prosthesis to contribute with additional data for comparing DC with ML control as proposed by Resnik et al*. *[6].

## Methods

### Participants

The study was approved by the ethics committee of the University Medical Center Groningen (METc 2018.268). To be eligible for inclusion the participants had to meet the following criteria: adults with unilateral upper limb absence at the transradial or wrist level. Both individuals with an upper limb deficiency caused by amputation or congenital deficiency were included. Individuals had to use DC. All participants gave written informed consent before the start of the first session. After training was completed, participants were awarded a gift voucher for their participation.

### Prosthesis and Socket

This study used the Michelangelo Hand (Otto Bock, Germany, Ref: 8E500 = R-M) which was fitted with a custom wrist flexion/extension unit and a wrist rotation unit (AxonRotation, Otto Bock, Germany, Ref: 9S503). The Michelangelo hand could perform open hand, fine pinch, lateral pinch, wrist rotation and wrist flexion/extension.

**Fig. 1:**
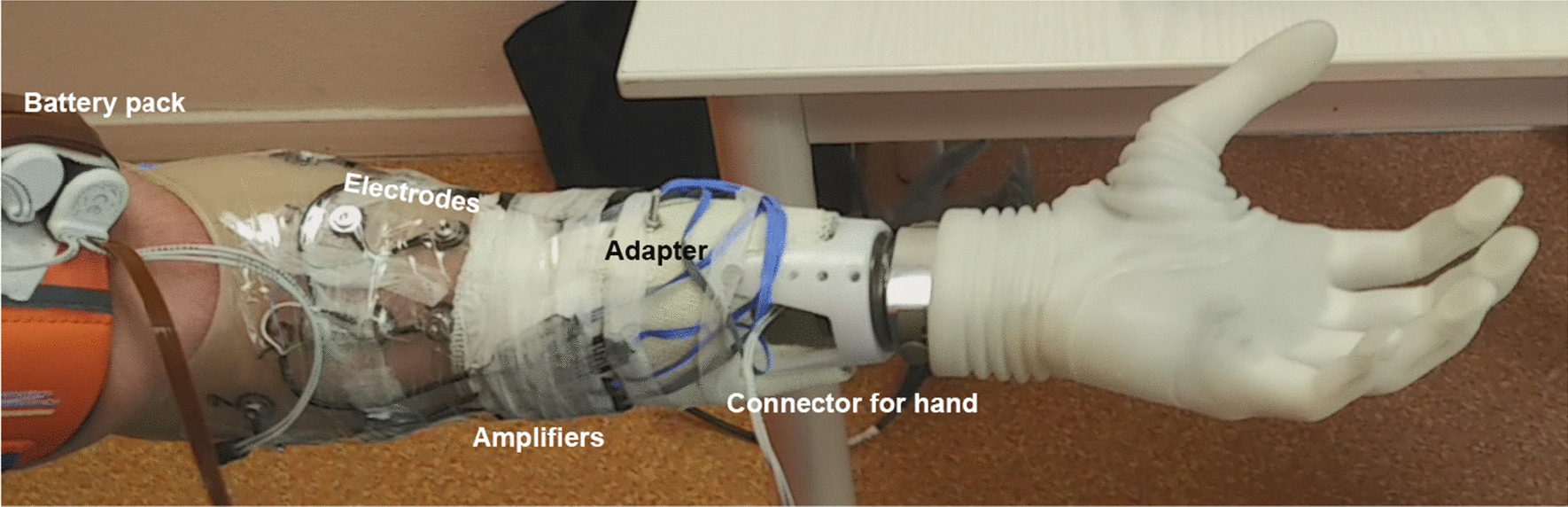
An example of the complete prosthetic setup as used in the study attached to a left arm. While all participants used the same setup, the positioning of the amplifiers and adapter differed slightly between participants to accommodate for a different anatomy

The sockets were 3D-printed using antibacterial thermoplastic (Otto Bock, Germany, Ref: 616T269) based on a 3D scan of the participant’s stump. The printed socket was adjusted by a certified orthopaedic technician for optimal fit. A connector for the Michelangelo hand was attached to the socket using a bespoke adapter made in Cellacast (Lohmann & Rauscher, Germany, Ref 25 202). Furthermore, eight bipolar Otto Bock Myo plus Electrodes with amplifiers (Otto Bock, Austria, ref: 13E401 = G140-60) were placed equidistantly (i.e. not targeted) around the socket using a numbered positioning band (Otto Bock, Austria, Ref: 623F50) according to the manufacturer’s specification [24] avoiding the location corresponding to the ulnar bone. The battery pack was strapped on the upper arm using an adjustable Velcro band placed proximal from the elbow joint. See Fig. 1.

The socket and the battery pack were worn during training, but the Michelangelo hand was only worn during functional training (see section Functional Training) and when performing the pre/post-test (see "Pre/post-test").

### Machine learning

The ML control system consisted of three components: (1) feature extraction, (2) regression between EMG features and hand movement commands, (3) post-processing of movement commands to improve usability and suppress possible errors.

To compute features, the eight-channel raw EMG data were windowed into overlapping windows of 128 ms with a step size of 50 ms; in a real-time application this allows to transmit control commands at 20 Hz with less than 200 ms delay. Hudgins-style time-domain features [25], namely the framewise mean absolute value, zero crossing rate, waveform length, and slope sign change, were computed and z-normalized before further processing.

A feed-forward neural network was used as a regressor to compute hand movement commands from the pre-processed EMG data. The neural network used two fully connected hidden layers with 50 and 25 neurons respectively, each followed by a tanh nonlinearity, and a final linear layer with as many output neurons as hand movements. Thus, for each window of EMG data, a vector with seven nonnegative elements, corresponding to the strengths of the seven different movements (fine pinch, lateral pinch, wrist rotation clockwise, wrist rotation counter-clockwise, wrist flexion, wrist extension, hand open) was computed. This vector is converted to a new vector of four elements whose values range from − 100 to + 100 and represent the four DoFs/grips, where opposing DoFs/grips (e.g. wrist flexion and wrist extension or fine/lateral pinch) are combined into a single value, where the opposite movements are represented by positive or negative values respectively. In the case that the elements representing fine/lateral pinch and hand open both differed from zero, the element with the lowest absolute value was discarded as the prosthetic hand cannot close in a pinch and open at the same time. The neural network was trained on mini-batches of 64 EMG windows using a mean squared error regression loss and the ADAM optimizer [26], with early stopping on a randomly chosen validation set of 10% of the entire training data.

Training data for the algorithm were recorded using the following EMG recording procedure. For each movement, the maximum voluntary contraction was performed to establish a baseline. Following baseline measures, each movement was recorded three times, where the participant was asked to follow a trapezoidal reference line [27] with a maximum of 30%, 60% and 90% maximum voluntary contraction for five seconds. During this recording, the contraction force was estimated from the real-time EMG activity and plotted on a screen visible to the subject together with the reference force, thus providing biofeedback to the subject. Over the course of user training, the EMG recording procedure was repeated several times, as described below.

Initially, hand open and fine pinch were trained, thus making the control comparable to DC and making the initial use of the system easier for the user. As participants progressed through the training scheme, more movements were added, namely whenever both the participant and the experimenter considered control to be robust using a qualitative assessment. Apart from the pre-training (see "Pre-training") the algorithm was always trained on data from the most recent five EMG recording procedures. Five was chosen as a compromise in order to supply the algorithm with sufficient data and at the same time account for changes in the EMG patterns resulting from user training. The EMG recording procedures were performed with the arm in one of three postures: hanging down by the side, resting on the armrest of the chair or reaching in front with the arm stretched out. At least one set of data from each posture was used to train the algorithm.

A moving average post-processing filter was implemented, where the average was taken over the last four regression outputs; this setup was found useful to suppress recognition outliers and provide a smoother hand control.

### Study design

**Fig. 2:**
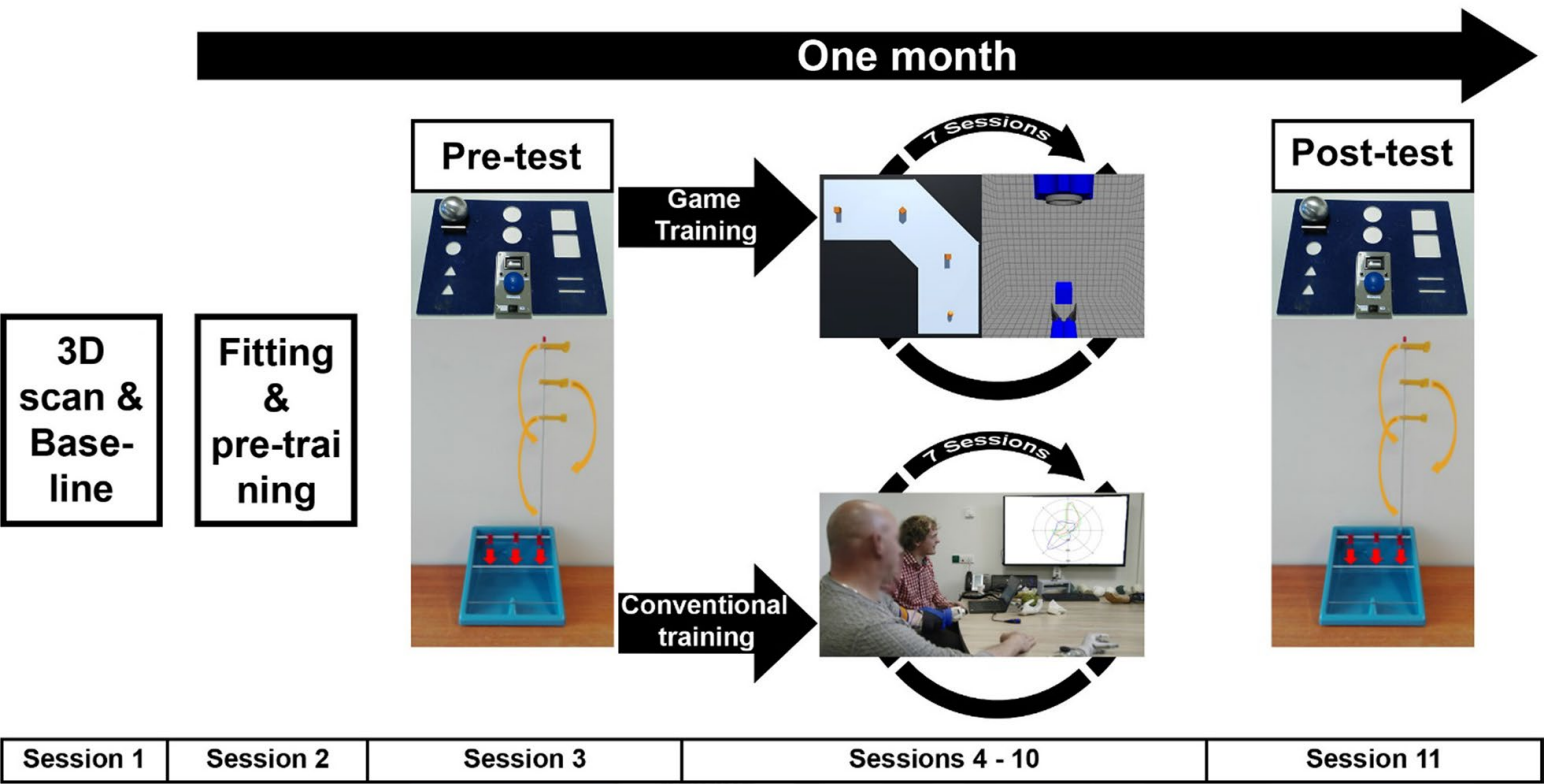
Overview over the sessions

Participants were randomly assigned to either the game training group or the conventional training group using a computer program. Participants in both groups followed the same training scheme, but with different content during training. The scheme consisted of 11 sessions. In the first session the stump of the participant was scanned, so a 3D-printed socket could be made (see Materials) and the baseline measure was performed (see Baseline). In the 2nd session the participant performed pre-training (see Pre-training). In the third session, the pre-test was performed (see "Pre/post-test"). Session 4–10 were training sessions (see Training). The last session was a post-test that was identical to the pre-test. Pre-training (session 2) and post-test (session 11) were scheduled within one month leading to two to three training sessions per week, see Fig. 2. This scheme was a compromise between having enough sessions to detect a difference and making recruitment of participants feasible [28, 29].

### Baseline measurement

The participant performed the baseline measurement using their own DC prosthesis. The baseline consisted of two parts; the spherical subset of the Southampton Hand Assessment Procedure (SHAP) [30] and The Clothespin Relocation Test (CRT) [31, 32]. The spherical subset of the SHAP consists of 4 tasks: moving the light sphere, moving the heavy sphere, pouring water from a carton, and opening a jar. The time for each SHAP task was recorded and converted to a score between 0 and 100 where 100 represents able-bodied performance. We used the method described by Burgerhof et al. to calculate the SHAP score [33]. The CRT was setup with 6 pins, 3 yellow pins (resistance 4.44 N) placed on the vertical bar and 3 red pins (resistance 8.89 N) placed on the horizontal bar. Participants were asked to move one red pin to the other horizontal bar, then move a yellow pin down the vertical bar followed by another red pin etc. The time to move all clothespins and the number of dropped clothespins were recorded. The participant was not given a time penalty for dropping pins.

### Pre-training

Pre-training started by briefly introducing the participant to ML control and how it differs from DC. Pre-training differed depending on group allocation, either the game training group or the conventional training group. Participants in the game training group trained using the MyoBox game (see game training group) for ten minutes, followed by three EMG recording procedures for open hand and fine pinch. Participants in the conventional training group trained with a coach for ten minutes to find two phantom movements or muscle contractions that could be used to activate the open hand and fine pinch commands of the prosthesis. After the ten minutes with the coach, the participant performed three EMG recording procedures for open hand and fine pinch.

At the end of pre-training for participants in both groups, the Michelangelo hand was connected to the socket. The participant was then given five minutes in which they could get acquainted with the hand and the control.

### Pre/post-test

Before starting the pre-test and post-test, two EMG recording procedures were performed to get ‘fresh’ data. The movements recorded before the pre-test were the same as in the pre-training. Data from the preceding five EMG recording procedures were combined to train the ML algorithm.

The contents of the pre-test and post-test were identical and consisted of the SHAP and CRT as in the baseline measurements. The test order was randomised between participants but was kept the same for each participant between the pre- and post-test.

### User training

User training sessions lasted between 45 and 60 min depending on the amount of rest needed by the participant. The participant could rest between each part of the training if needed. Initially, training focused on adding wrist rotation in addition to hand open and fine pinch. When the participant had good control over these two DoFs, lateral pinch would be added and lastly wrist flexion/extension. At the end of each training session the participant performed five minutes of functional training (see "Functional training").

### Game training group

The game training group trained using two games named MyoBox and Prosthesis Gripper. Myobox was designed to maximise separation of EMG patterns [15] and Prosthesis Gripper was designed to train functional prosthesis use. Prosthesis Gripper was originally designed for DC [19, 34], but was adapted for ML control. In each session, participants played both games for approximately 10 min.

**Fig. 3:**
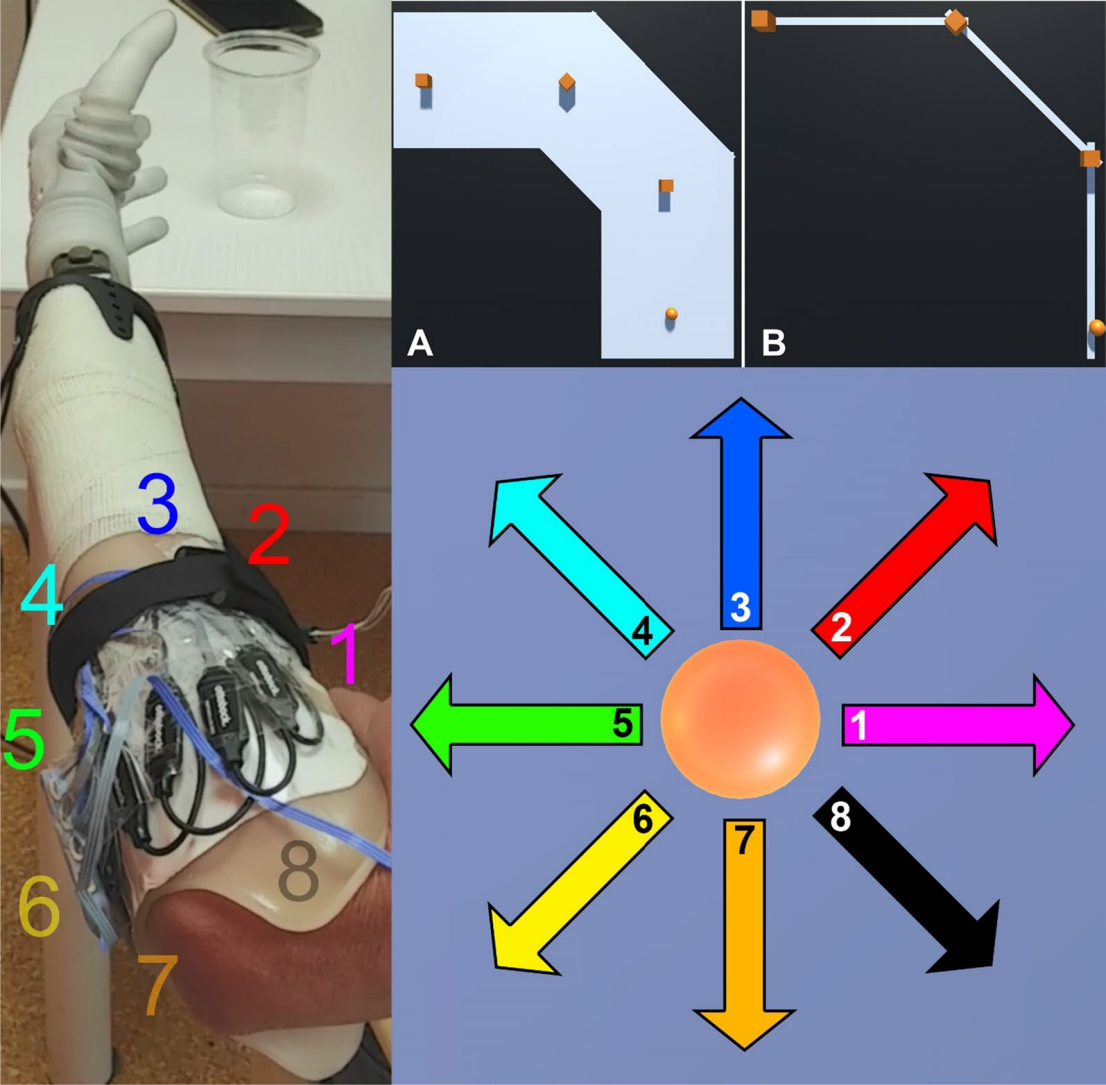
Mapping of RMS to avatar control. Left: placement of electrodes on a left arm, with electrode 1 corresponding to RMS_1_, electrode 2 corresponding with RMS_2_ etc. Upper right, A: First orientation of Myobox. The player controls the orange ball at the bottom and is tasked with collecting the three orange boxes while staying on the light blue platform. Upper right, B: First orientation of Myobox with the smallest possible platform size in the game. Lower right: directions of the avatar. The colour and the number inside of the arrows correspond with the coloured electrode number on the left-hand side of the figure

In MyoBox players controlled a ball which functioned as the game avatar using EMG. The goal of the game was to collect boxes by hitting them with the ball while staying on the platforms, see Fig. 3 upper right. The ball was controlled using a direct mapping between the root mean square (RMS) of the EMG of each electrode and avatar direction, see Fig. 3. The direction was calculated as:

1 $$y=\frac{RM{S}_{2}+RM{S}_{3}+RM{S}_{4}}{3}-\frac{RM{S}_{6}+RM{S}_{7}+RM{S}_{8}}{3}$$

2 $$x=\frac{RM{S}_{1}+RM{S}_{2}+RM{S}_{8}}{3}-\frac{RM{S}_{4}+RM{S}_{5}+RM{S}_{6}}{3}$$

where y is the vertical direction and x is the horizontal direction. $RM{S}_{x}$ corresponds to the arrows and numbers in Fig. 3.

To obtain the goal in Myobox, players had to explore different muscle contractions and learn which muscle contractions made the ball move in the desired direction. As the participant learned the control and progressed through MyoBox, the game became more difficult by decreasing the platform width. In this way, participants had to control the ball with higher precision to avoid falling off the platform, see Fig. 3 upper right. Since participants would learn to control the ball in the game without initially connecting the muscle contractions they learn to the actuation of the prosthesis, this mapping strategy might be unintuitive at first. However, it has been shown to be potentially more robust [15] and consequently the risk that the neural network makes a misclassification might be smaller. As a result of the mapping, the EMG patterns generated by the muscle contractions used to control the ball in different directions were distinct. These patterns were later used to train the ML algorithm. For more details about MyoBox see also [15].

After playing MyoBox, an ML algorithm was trained using the muscle contractions learned by playing MyoBox. The real-time control output of the ML algorithm was then used to play the game Prosthesis Gripper. Prosthesis Gripper trained two aspects: proportional control and grasping.

Proportional control was trained using a tracking task, where the participant had to control the game avatar left or right. In the beginning of training when focus was on training wrist rotation, the muscle contractions corresponding to wrist rotation moved the avatar left and right. Similarly, when focus was on training wrist flexion/extension, the muscle contractions corresponding to wrist flexion/extension moved the avatar left and right. In the tracking task, participants controlled the avatar, so it followed a light that moved from the centre of the screen to one of the sides. When the avatar was close enough, a beam would show, and the participant was awarded points. If the avatar were too far away from the light, the participant would lose points. When the light reached the edge of the screen it would stop moving. When this happened, the participant had to get the avatar under the light for one second after which the light would disappear and be replaced by a dispenser indicating the start of training for grasping.

**Fig. 4:**
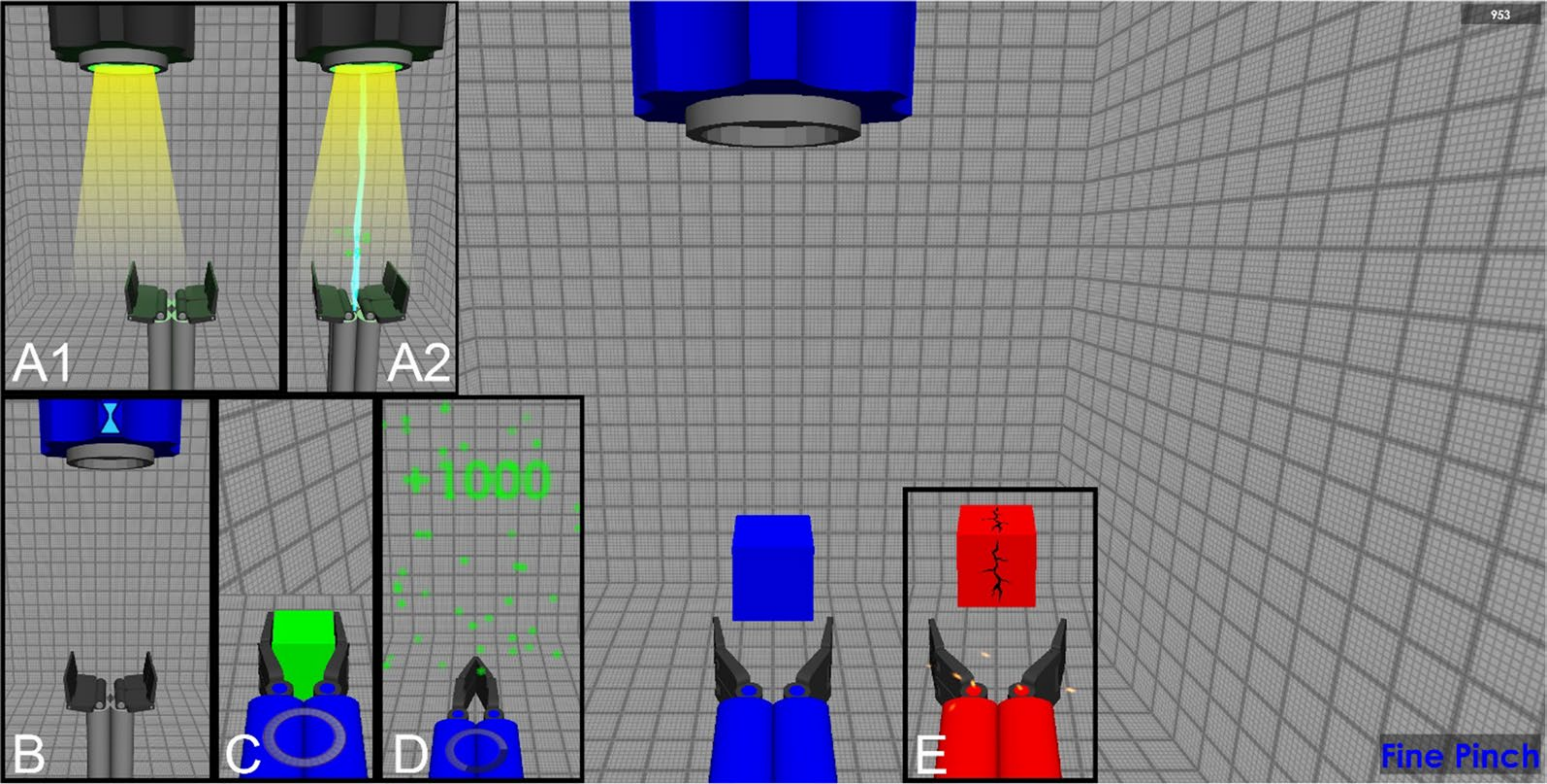
Prosthesis Gripper with inserts showing different game situations. Main screen shows the gripper about to grasp a blue box which corresponds to fine pinch. Bottom right corner shows the required grip. Top right corner shows the score, which was always visible. Insert A1, tracking task where the avatar must follow the light. Insert A2, a beam shows when the avatar is close enough and points are awarded and written on screen (green numbers). Insert B, after the tracking task is completed, the player must activate the grip corresponding to the colour of the dispenser at the top. This must be done before the hourglass shown on the dispenser runs out. Insert C, when the object has been grasped, it turns green and the player must release it again. Insert D, when the object is released, a thousand points is awarded. Insert E, if the gripper is opened too much, sparks showed, and the gripper would force close

Grasping was trained with a stationary game avatar being a gripper. First, the participant had to activate the grip corresponding to the colour of the dispenser at the top of the screen (blue for fine pinch, red for lateral pinch, see Fig. 4), within ten seconds. A grip was activated when the ML algorithm output for that DoF was ± 25 while all other DoFs were between 0 and ± 24. If the grip was not activated within ten seconds, the game would go back to proportional control training. When the grip was activated, the dispenser would drop an object. The object fell towards the gripper and the participant had to first open the gripper and then grip it using the correct grip based on the colour of the object. The gripper could maximally open up to 1.7 times the width of the object. If the gripper was opened more than 1.5 times the width of the object, sparks would show. If the gripper was fully opened, it would force close itself. This constraint was added to the game to avoid that the participants fully opened the gripper every time, as skilled prosthesis users match the opening of the hand to the size of the object they are grasping [35]. In addition, some of the objects were fragile (indicated with cracks) and when such an object was presented the participant had to be careful not to close the gripper too forcefully, otherwise the object would break. When an object was successfully grasped, the participant had to release it again to earn points and proceed to proportional control training.

### Conventional training group

**Fig. 5:**
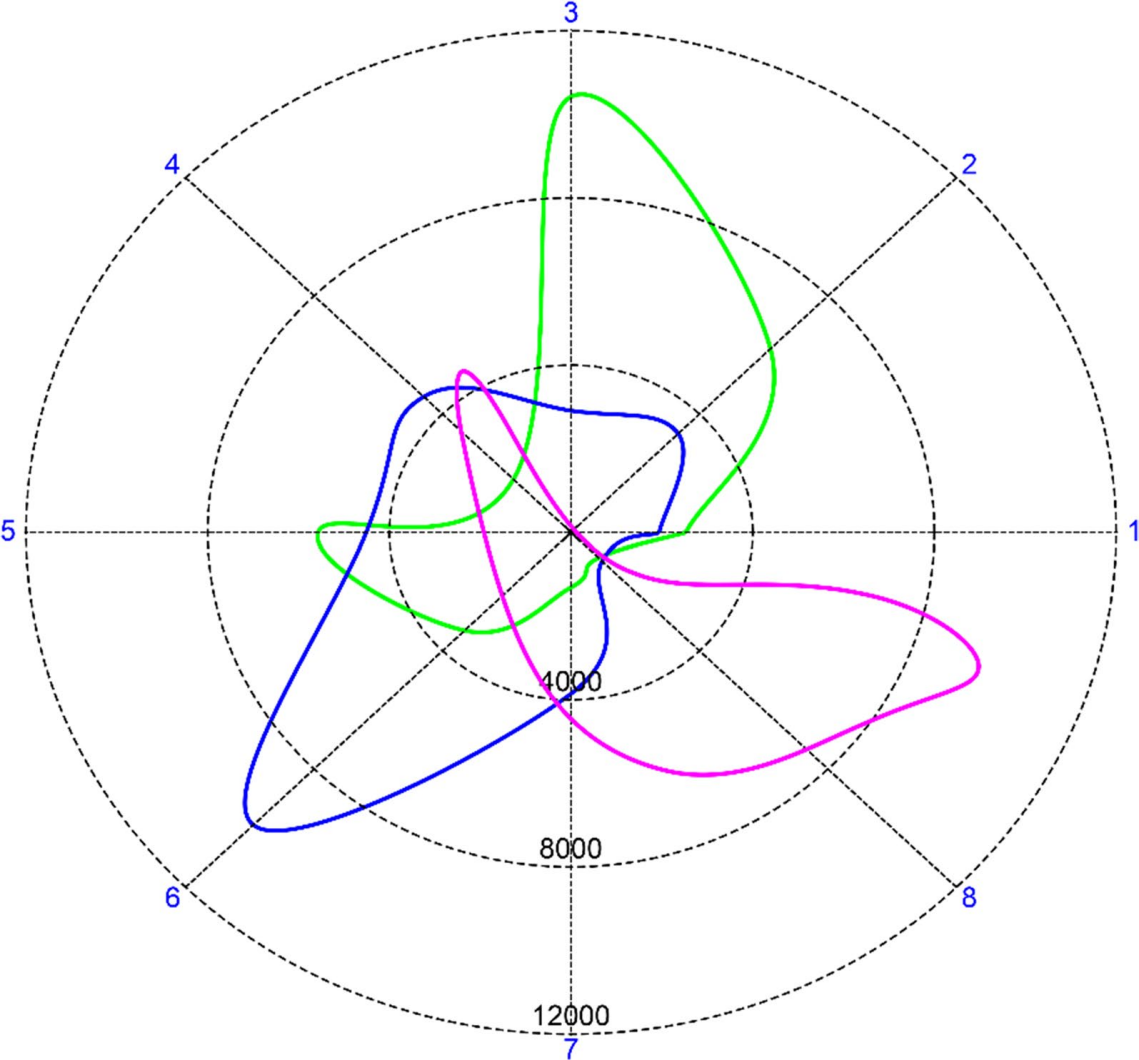
Example of the spider plot used in conventional training. The spider plot shows the RMS of the EMG for each channel and serves as a simplified representation of the feature space with each movement represented by one coloured shape. Possible overlap can be visually perused, and the coach can guide the participant to minimize it

Training for the conventional training group followed three steps: EMG recording procedure, two times Motion Test and coaching. The system training procedure was conducted as described in the Machine Learning section of this paper. The Motion Test [36] is a computer-based test designed to assess the control a participant has over the ML output, but in this study it was used as a training tool. In the Motion Test the participant conducted several movement trials. In each movement trial, the test prompted the participant with a movement at a specific force level that the participant had to perform. The participant had to perform the movement in such a way that the ML algorithm output was the highest for the prompted movement at the correct force level for two seconds within a six second duration window. The force level corresponded to the force levels used during the EMG recording i.e. 30%, 60% and 90% with a margin of 15%, 20%, and 30% respectively. The number of movement trials depended on the number of trained movements. Each trained movement resulted in three movement trials corresponding to the trained force levels. The Motion Test was performed two times in a row. After the second Motion Test, the participant received coaching from the experimenter. The coaching was based on a spider plot which showed a simplified representation of the feature space. The representation showed the RMS of the EMG patterns, see Fig. 5.

The coaching followed the work of Powell and Thakor [12]. The principle was to make EMG patterns as separate as possible by adapting the phantom movement of the participant. For participants with no phantom limb sensation, Powell and Thakor suggested that participants “mimic the muscle activity of their intact limb”. The coach asked the participant to perform variations of the phantom movements (muscle contractions) used for training. As an example, when performing a fine pinch, the index, ring and little finger can be either flexed or extended (or a combination thereof). When the participant performs the different variations of a fine pinch, the coach will observe the feature space as shown in Fig. 5 and determine which variation is most separate from the other movements. Coaching was given by two Dutch students in Human Movement Science (University of Groningen) who had been trained to this effect. Coaching was always done under supervision of either MBK or AWF.

**Fig. 6:**
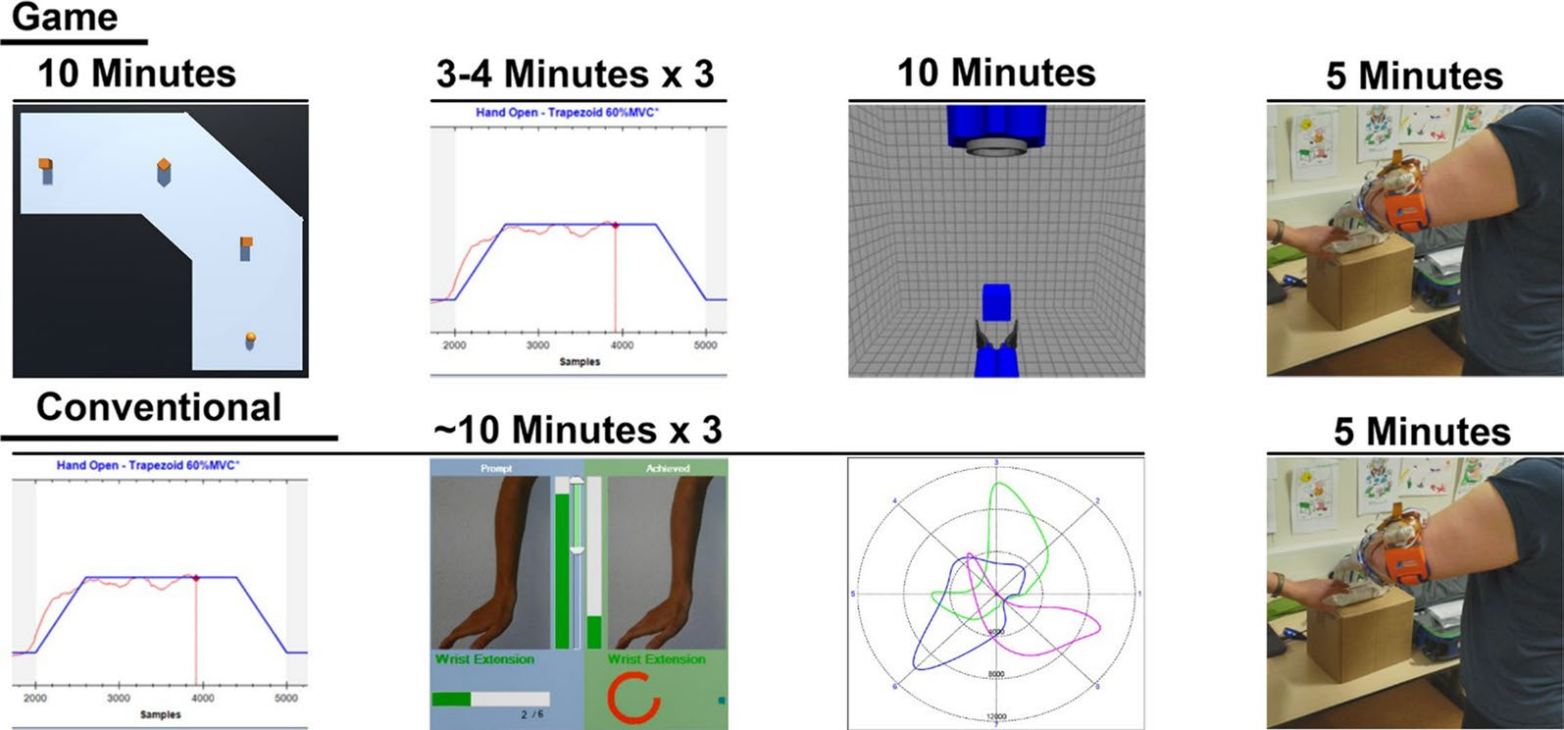
Overview of the training sessions for the training groups. Length of each activity is written above the pictograms. Top row: Game training starting with Myobox, followed by three times EMG recording, Prosthesis Gripper and ending with functional training. Bottom row: Conventional training starting with three times combined EMG recording, Motion Test and coaching and ending with functional training

In each session, the three steps i.e. EMG recording, two times Motion Test and coaching, were repeated three times. See Fig. 6 for an overview over both training groups.

### Functional training

Functional training consisted of grasping an object from a table and placing it on a box (height 21 cm) and grasping an object from the box and placing it on the table. Three objects were used; a plastic lid, a 0.5 L plastic bottle filled with water and a plastic single-use cup. The training protocol was:

1. Grasp bottle placed horizontally on the box, rotate and place vertically on the table
2. Grasp plastic lid placed vertically from the table, rotate and place horizontally on the box
3. Move plastic cup placed on the table to the box.

The protocol was repeated for 2.5 min while sitting in front of the table. After a break, the protocol was repeated for 2.5 min while standing in front of the table. The different objects and their orientations were chosen to promote the use of wrist rotation and the different grips. If participants tried to perform the protocol using only one grip and compensatory movements, they were encouraged to use wrist rotation or other grips.

### Data analysis

To answer the research questions related to the separability and consistency of EMG patterns metrics measuring these properties were calculated and reported for each participant. The separability metrics were the Inter-class Distance Nearest Neighbour (IDNN) and Inter-class Distance All Neighbours (IDAN). The consistency metric was the Within-class Distance (WD). We report the mean of each of these metrics per session. These three metrics were taken from [37]. See Appendix for further details.

To answer the research question related to the number of DoFs used, the number of DoFs used by each participant at the post-test was reported, since this differed from the pre-test.

To answer the research question related to functional outcomes, the scores of the SHAP and CRT were reported for each participant separately using the ML controlled prosthesis and the participants’ own DC prosthesis (baseline).

## Results

Eight participants were recruited, and four participants completed the training protocol, see Table 1. Participant P6 had to cancel his last training session and therefore trained one session less than the other participants. Four participants did not complete the training protocol due to poor socket fit, fatigue, or issues with scheduling appointments (Table 1).

**Table 1:** Characteristics of the participants

Acronym	Sex	Age	Acquired limb loss?		Own prosthesis	Group	Completed
P1	F	52	Yes	Conventional 1 DoF		Game	No. Was excluded before pre-test due to poor socket fit
P2	F	39	No	Conventional 1 DoF		Conventional	Yes
P3	M	59	No	Conventional 1 DoF		Game	Yes
P4	M	47	Yes	Conventional 1 DoF		Conventional	No. Dropped out during pre-test due to fatigue
P5	M	49	Yes	Multi-articulate multiple DoFs		Conventional	Yes
P6	M	56	Yes	Multi-articulate multiple DoFs		Game	Yes
P7	M	52	Yes	Conventional 1 DoF		Game	No. Dropped out after baseline measure due to scheduling conflicts
P8	M	74	Yes	Conventional 1 DoF		Conventional	No. Dropped out during baseline measure due to fatigue

*M* male, *F* female, *DoF* degree of freedom, *DoFs* degrees of freedom, *P* participant number

### EMG pattern separability and consistency

**Fig. 7:**
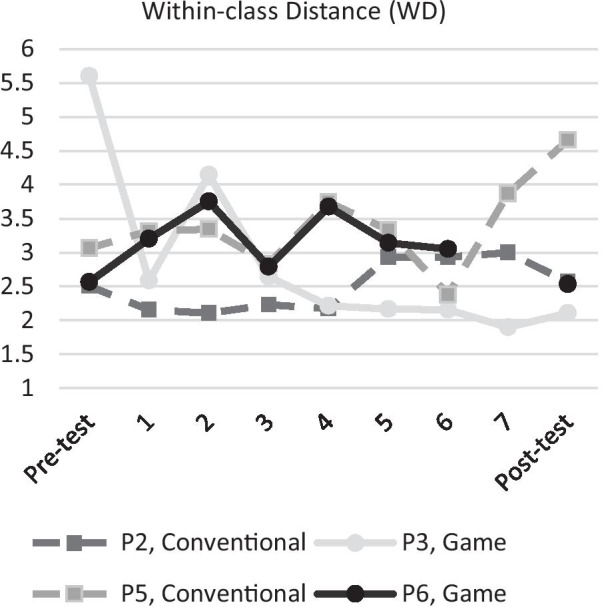
Within-class Disstance (WD) per participant. Lower WD indicates better performance. Participants in the game group are plotted in solid colour, while participants in the conventional group are plotted with dashed lines. Participant P6 had to cancel his last training session (session 7) so participant P6 has no data for this session. All sessions except for the Pre-test used two DoFs

**Fig. 8:**
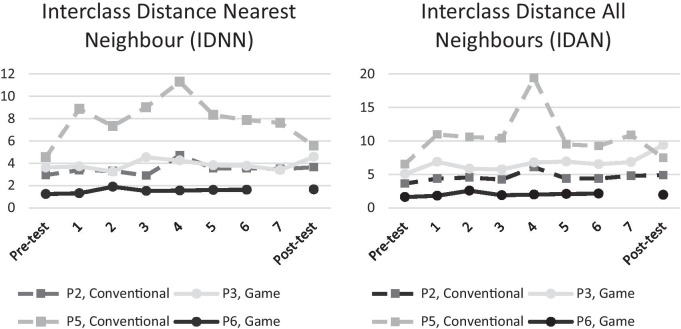
Left: Inter-class Distance Nearest Neighbour (IDNN) per participant. Right: Inter-class Distance All Neighbours (IDAN) per participant. Higher IDNN/IDAN indicate better performance. Participants in the game group are plotted in solid colour, while participants in the conventional group are plotted with dashed lines. Participant P6 had to cancel his last training session (session 7) so participant P6 has no data for this session. All sessions except for the Pre-test used two DoFs

To answer research questions 1 and 2 related to EMG pattern separability and consistency we plotted the EMG metrics calculated from the pre-test, each training session and post-test in Figs. 7, 8. Figure 7 shows the WD, which is a measure of the consistency of the EMG patterns where lower WD corresponds to more consistent EMG patterns, for individual participants. Most participants had erratic changes in WD except for participant P3 (game) who after the second training session showed a steady reduction in WD. On the group level there appear to be no clear difference between both groups in terms of reduction of WD or rate of improvement.

Figure 8 shows the IDNN and IDAN metrics which measure the separability of EMG patterns. Higher IDNN and IDAN correspond to more separable EMG patterns. Most participants showed a minor increase in IDNN and IDAN except for participant P5 (conventional) who achieved high IDNN and IDAN during training until the post-test.

### Functional outcomes

**Fig. 9:**
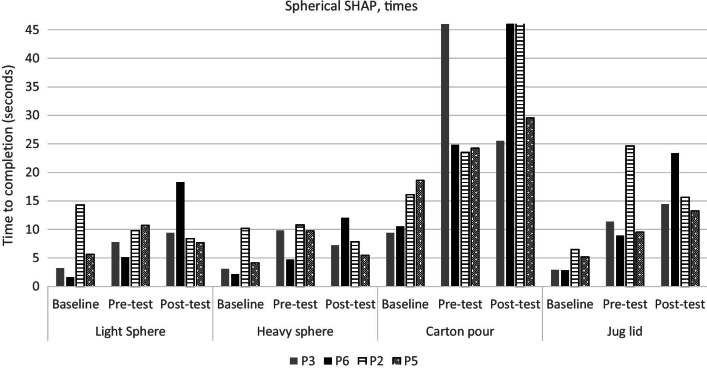
Times for completing each of the four SHAP tasks per participant. Participants in the game group are in solid colour, while participants in the conventional group are shaded. Baseline was conducted before training using the participants’ own DC prosthesis. Pre- and post-test was conducted using the Michelangelo hand controlled with ML. In the pre-test all participants used one DoF and in the post-test participants used two DoFs

**Fig. 10:**
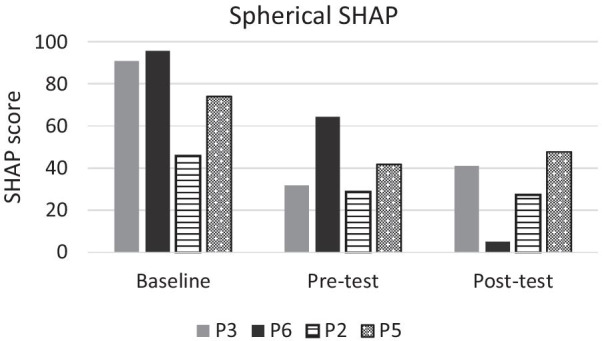
SHAP scores for the spherical subset of the SHAP per participant. Higher scores reflect better performance. Participants in the game group are in solid colour (P3, P6), while participants in the conventional group are shaded (P2, P5). Baseline was conducted before training using the participants’ own DC controlled prosthesis. Pre- and post-tests were conducted using the Michelangelo hand controlled with ML. In the pre-test all participants used one DoF and in the post-test participants used two DoFs

**Fig. 11:**
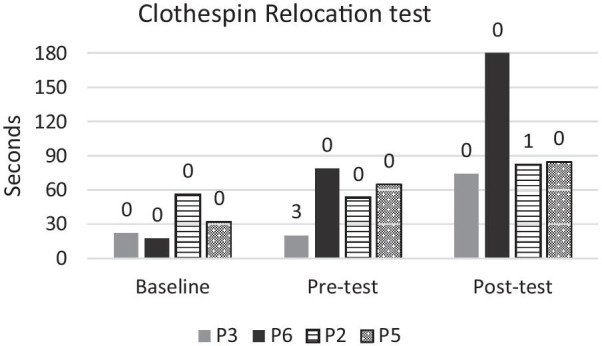
Times for completing the clothespin relocation test per participant. Lower time scores indicate better performance. The number written above each bar indicates how many pins were dropped (maximum = 6). Participants in the game group are in solid colour (P3, P6), while participants in the conventional group are shaded (P2, P5). Baseline was conducted before training using the participants’ own DC controlled prosthesis. Pre- and post-tests were conducted using the Michelangelo hand controlled with ML. In the pre-test all participants used one DoF and in the post-test participants used two DoFs. Participant P3 was unable to grasp any of the yellow clothespins at the pre-test, so the time is for the red clothespins only

The results from the spherical subset of the SHAP are shown in Figs. 9, 10 and the results from the CRT are given in Fig. 11. Training in either group did not appear to have improved performance in a consistent manner. Furthermore, baseline measurements with the user’s own prosthesis appeared to be consistently superior to either test using ML. Note that all participants controlled one DoF at the pre-test and two DoFs at the post-test.

## Discussion

The aim of this explorative study was to assess if game-based training leads to (1) more separated and consistent EMG patterns, (2) a higher rate of improvement in EMG pattern separability and consistency as result of learning, (3) use of more degrees of freedom and 4) better outcomes in functional prosthesis use, compared to conventional training with coaching.

### Separability and consistency of EMG patterns

In terms of EMG separability, participants P2, P3 and P5 all show a trend towards higher separability, but the increase was not very consistent over the training sessions. Especially participant P5 showed a major increase in separability during training, but at the post-test his separability was almost the same as it was at the pre-test. For participant P3, the increase in IDAN was promising for game-based training, as an increase in IDAN was also found in able-bodied participants who trained using games [15]. Game training using movements that do not match the actuation of the prosthesis (e.g. prosthetic fine pinch is not activated by phantom fine pinch) can lead to more separable EMG patterns. A similar strategy could also be applied to conventional training, but it might have limited efficacy due to the internal focus of attention and the declarative statements of the coach.

In terms of EMG consistency, only participant P3 had a consistent decrease (lower is better) from the 2nd training session. From these results we see no indication that game-based training could lead to more separated and consistent EMG patterns. Furthermore, we see no indication that game-based training could lead to a higher rate of improvement in EMG pattern separability and consistency. However, neither conventional nor game training seemed to lead to consistent improvements which might be due to the number of training sessions (discussed later) or the robustness of ML control. If ML control is not robust, participants will hardly improve but instead spend their training time adapting to the system. From this perspective, it might be better to not retrain the ML algorithm as often as it was done in this study, and instead have the participant spend more time learning the peculiarities of a trained ML algorithm for a longer period of time. However, this also means that performance will be limited by the quality of the trained ML algorithm and a ML algorithm that is not well trained might be unusable. Future research should investigate how learning is affected by keeping the ML algorithm (near) constant and compare with a ML algorithm that is retrained at every session.

### Use of more degrees of freedom

All participants controlled two DoFs at the post-test. From these results we see no indication that game training could lead to the use of more DoFs than conventional training. This can however be expected given our results that game training did not seem to lead to more separable EMG patterns.

None of the participants was sufficiently skilled at controlling two DoFs at the time the training was completed, so adding the third DoF was not considered. From our experience in running the experiment, we consider it more important that participants have robust control of a few DoFs, than adding additional DoFs that might deteriorate control. However, we would argue that in the long run users benefit from the use of more DoFs.

### Functional outcomes

Functional outcomes did not seem to improve as a result of training for either group. Therefore, we see no indication that game training leads to better functional outcomes. It is important to underline that the post-test was performed using an extra DoF (compared to the pre-test) which might have reduced the robustness of the control. We observed that some participants prioritised using the additional DoF over completing the tasks as fast as possible. While participants knew that the outcome of the tasks was time, they also were encouraged to utilise both DoFs during functional training. It can be hypothesized that participants who used two DoFs might have had a reduced amount of compensatory movements. Compensatory movements can lead to overuse resulting in pain and reduced mobility [38–40]. Reducing compensatory movements might therefore be a more important goal than reducing completion times in functional tasks. Outcome measures evaluating the movement quality should therefore be part of future studies.

### Length of training

To our surprise, training did not seem to lead to consistent improvements in terms of EMG patterns nor led to improved performance in functional tests. Comparable results were obtained in the study by Resnik et al. [6] where improvements in the Jebsen Taylor Hand Function page turning test showed erratic behaviour. This is in contrast with the study by Kuiken et al. [4] where training led to improved functional performance. However, the participants in the study of Kuiken et al. trained for at least four hours prior to a one-month home trial which is considerably more than in the current study and in the study by Resnik et al. It is likely that with additional training and use, participants in our study would have improved and eventually achieved better performance with ML control. We suggest future studies include more training and home training in order for participants to achieve good control.

### Comparing direct control and machine learning control

To provide additional data for comparing DC with ML control, we performed baseline measurements as proposed by Resnik et al*. *[6] using our participants’ own DC prostheses. Similarly, to Resnik et al*.* we found that DC control seems to outperform ML control. However, it should be noted that all participants in our study had several years of experience using their own DC prosthesis and were naïve to both the prosthesis hand used in the current study as well as ML control. Therefore, it is possible that with sufficient training participants would have been able to achieve better performance with ML control than with DC. A study by Kuiken et al*.* supports this claim [4]. In their study, they showed that after one month of home use, participants tended to have better performance using ML control than DC. Future research should investigate longer training periods and/or home trials using the same prosthesis and control algorithm. A serious game as proposed in this study might be beneficial for such studies as participants can train at home without supervision using such games.

### Recommendations for future studies

During this study we learned several new things we believe are helpful for future training studies using ML control. Firstly, pre-training before performance assessments ensures that participants have some basic control and understand the basic concepts of ML control. Secondly, the importance of the socket fit should not be underestimated. Any non-ideal fit in terms of user comfort or electrode–skin contact will likely have a drastic negative impact on control performance. In pilot testing we had used sockets made of plaster under the guidance of an orthopaedic technician and while initial results were promising, the plaster sockets were not robust enough when applying weight to the prosthesis which led to noise in the EMG and discomfort. We therefore advise any research group to manufacture professional sockets in trials with prolonged (home) use. Thirdly, increasing DoFs gradually appeared to be a good way to ease participants into ML control and made the transition from DC to ML control easier. Lastly, including functional training as part of the training scheme gave participants the opportunity to learn how external factors like weight and posture affects control which helped them to adapt.

### Limitations

The main limitations of this study are the limited sample size and the number of training sessions. While we recruited eight participants, half of them dropped out of the study for various reasons. Unfortunately, due to limited resources, we were unable to find new participants to replace those that dropped out of the study. In terms of the number of training sessions, we made a trade-off between resources, providing sufficient training and making participant recruitment feasible. We believe recruiting participants will become more difficult if more training sessions are added, but from our results it is also evident that additional training sessions are needed. We suggest that future research combines training sessions with a lengthy home trial. In our case, a home-trial was not feasible due to time constraints, limited financial resources, hardware availability and safety issues. Given that there are now at least two commercial ML control systems on the market in Europe and in the US, it should be feasible in future research to conduct a lengthy home-trial. Finally, our study only provides quantitative data which does not capture the users’ satisfaction with the training scheme and the control. Such data could be measured using qualitative methods such as questionnaires which might reveal a preference for either control method which is not directly related to the functional performance we captured.

## Conclusions

To conclude, we have shown that serious games might be used for ML control training with the target population, as we found similar results in the game group compared to the conventional group. In addition, we found no consistent improvement in terms of EMG separability and consistency and no improvements in functional outcomes. However, control of two DoFs might require more training than what was provided in this study and with more training improvements in EMG metrics and functional outcomes might improve. Furthermore, we have provided additional data and knowledge regarding ML control versus DC which adds to the data from previous research by Resnik et al. and Kuiken et al*.* Research efforts should be combined to reach larger sample sizes in order to provide more evidence for different training schemes.

## Data Availability

The datasets used and/or analysed during the current study are available from the corresponding author on reasonable request.

## Appendix

### Calculation of EMG metrics

We define the Within-class Distance (WD) as:

3 $$WD=\frac{1}{m}\sum_{j=1}^{m}\left(\sum_{k=1}^{3}\frac{{dist}_{kj}^{rj}*{dist}_{rj}^{kj}}{{dist}_{kj}^{rj}+{dist}_{rj}^{kj}}\right)$$

where m is the number of movements trained and ${dist}_{kj}^{rj}$ and ${dist}_{rj}^{kj}$ are half the Mahalanobis distances in feature space between the EMG patterns of repetitions r and k of movement j and between repetitions k and r of movement j respectively:

4 $${dist}_{kj}^{rj}=\frac{1}{2}\sqrt{{\left({\mu }_{Trj}-{\mu }_{Tkj}\right)}^{T}*{{S}_{Trj}}^{-1}*\left({\mu }_{Trj}-{\mu }_{Tkj}\right)}$$

5 $${dist}_{rj}^{kj}=\frac{1}{2}\sqrt{{\left({\mu }_{Tkj}-{\mu }_{Trj}\right)}^{T}*{{S}_{Tkj}}^{-1}*\left({\mu }_{Tkj}-{\mu }_{Trj}\right)}$$

We define Inter-class Distance Nearest Neighbour (IDNN) and Inter-class Distance All Neighbours (IDAN) as:

6 $$IDNN=\frac{1}{m}\sum_{j=1}^{m}\left(\underset{i=1,\dots j-1,j+1,\dots m}{\mathrm{min}}\frac{{dist}_{j}^{i}*{dist}_{i}^{j}}{{dist}_{j}^{i}+{dist}_{i}^{j}}\right)$$

7 $$IDAN=\frac{1}{m}\sum_{i=1}^{m}\left(\frac{{dist}_{j}^{i}*{dist}_{i}^{j}}{{dist}_{j}^{i}+{dist}_{i}^{j}}\right)$$

where m is the number of movements trained and ${dist}_{j}^{i}$ and ${dist}_{i}^{j}$ is half the Mahalanobis distance in feature space between movements i and j and between movements j and i of movement j respectively

8 $${dist}_{j}^{i}=\frac{1}{2}\sqrt{{\left({\mu }_{Ti}-{\mu }_{Tj}\right)}^{T}*{{S}_{Ti}}^{-1}*\left({\mu }_{Ti}-{\mu }_{Tj}\right)}$$

9 $${dist}_{i}^{j}=\frac{1}{2}\sqrt{{\left({\mu }_{Tj}-{\mu }_{Ti}\right)}^{T}*{{S}_{Tj}}^{-1}*\left({\mu }_{Tj}-{\mu }_{Ti}\right)}$$

## Abbreviations

DC: Direct control
DoF: Degree of freedom
DoFs: Degrees of freedom
EMG: Electromyography
IDAN: Inter-class distance all neighbors
IDNN: Inter-class distance nearest neighbor
ML: Machine learning
RMS: Root mean square
WD: Within-class distance
